# Prognostic impact of mRNA levels of osteopontin splice variants in soft tissue sarcoma patients

**DOI:** 10.1186/1471-2407-12-131

**Published:** 2012-04-02

**Authors:** Antje Hahnel, Henri Wichmann, Thomas Greither, Matthias Kappler, Peter Würl, Matthias Kotzsch, Helge Taubert, Dirk Vordermark, Matthias Bache

**Affiliations:** 1 Department of Radiotherapy, Martin-Luther-University of Halle-Wittenberg, Dryanderstr.4, Halle (Saale) 06110, Germany; 2 Centre for Reproductive Medicine and Andrology, Martin-Luther-University of Halle-Wittenberg, Ernst-Grube-Str. 40, Halle (Saale) 06907, Germany; 3 Department of Oral and Maxillofacial Plastic Surgery, Martin-Luther-University of Halle-Wittenberg, Ernst-Grube-Str. 40, Halle (Saale) 06907, Germany; 4 Department of General and Visceral Surgery, Diakoniekrankenhaus, Advokatenweg 1, Halle (Saale) 06114, Germany; 5 Institute of Pathology, Dresden University of Technology, Fetscherstr.74, Dresden 01307, Germany; 6 Clinic of Urology, FA University Hospital Erlangen, Glückstr. 6, Erlangen 91054, Germany; 7 Nikolaus-Fiebiger-Center for Molecular Medicine, FA University Erlangen-Nürnberg, Erlangen-Nürnberg, Germany

## Abstract

**Background:**

It is well known that osteopontin (OPN) plays an important role in tumor progression and that a high OPN expression level in several tumor entities correlates with poor prognosis in cancer patients. However, little is known about the prognostic relevance of the OPN mRNA splice variants.

**Methods:**

We analyzed the mRNA expression levels of different OPN splice variants in tumor tissue of 124 soft tissue sarcoma (STS) patients. Quantitative real-time PCR (qRT-PCR) was used to analyze the mRNA expression level of three OPN splice variants (OPN-a, -b and -c).

**Results:**

The multivariate Cox's proportional hazard regression model revealed that high mRNA expression levels of OPN splice variants are significantly associated with poor prognosis in STS patients (n = 124). Women (n = 68) with high mRNA expression levels of OPN-a and OPN-b have an especially elevated risk of tumor-related death (OPN-a: RR = 3.0, P = 0.01, CI = 1.3-6.8; OPN-b: RR = 3.4, P = 0.01, CI = 1.4-8.2). In particular, we found that high mRNA expression levels of OPN-b and OPN-c correlated with a high risk of tumor-related death in STS patients that received radiotherapy (n = 52; OPN-b: RR = 10.3, P < 0.01, CI = 2.0-53.7; OPN-c: RR = 11.4, P < 0.01, CI = 2.2-59.3).

**Conclusion:**

Our study shows that elevated mRNA expression levels of OPN splice variants are negative prognostic and predictive markers for STS patients. Further studies are needed to clarify the impact of the OPN splice variants on prognosis.

## Background

Osteopontin is a secreted phosphoprotein that plays an important role in tumor progression. It affects processes such as cellular growth, cell migration, invasion, metastasis and decay of the extracellular matrix [1]. Several studies showed that an increased OPN expression correlates with poor prognosis in cancer patients [2-4]. However, only a few studies have analyzed the importance of OPN for tumor progression in sarcomas. OPN protein expression level was shown to be elevated in tumor cells, and high OPN levels were associated with high tumor stage, tumor grade and poor survival in sarcoma patients [5-7]. Additionally, Bramwell *et al. *(2005) showed that OPN mRNA is also overexpressed in the tumor tissue of STS patients. In a previous study, we demonstrated that higher OPN protein expression levels in tumor tissue and serum were associated with worse prognosis in STS patients [8].

Young *et al. *(1990) identified three splice variants of OPN, but their specific functions remained unclear. In addition to the full-length form osteopontin-a (OPN-a) with 7 exons, there are the splice variants osteopontin-b (OPN-b) and osteopontin-c (OPN-c) with deletions of exon 5 and exon 4, respectively. The first in vitro studies demonstrated a different impact of the OPN splice variants on cell migration, invasion, apoptosis and proliferation in various cancer cell lines [9-13]. Compared to normal tissue, the tumor tissues of different cancer entities express higher levels of OPN splice variants [10,12,14-18]. However, only one study, which investigates breast cancer patients, analyzed the prognostic impact of the OPN splice variants [15]. In the present study, we analyzed the prognostic relevance of the expression level of the three OPN splice variants (OPN-a, -b and -c) in the tumor tissue of 124 STS patients.

## Methods

### Patients and tissue samples

We analyzed the frozen primary tumor samples of 124 STS patients and surrounding tissue of 15 STS patients by qRT-PCR for the mRNA expression of OPN splice variants (partially described in [8]). The clinical and histomorphological parameters of the STS patients are shown in Table 1. In total, 50.8% of STS patients (n = 63) were still alive after an average follow-up time of 57.3 (range 9-198) months, and 49.2% of STS patients (n = 61) died from tumor-related reasons after an average of 28.0 (range 2-201) months. The tumors were staged according to the Union for International Cancer Control (UICC) system. All patients gave their written informed consent to the Institute of Pathology, University of Halle, Germany, and Department of Surgery 1, University of Leipzig, Germany. The study was approved by the Ethics Committee of the Medical Faculty of the Martin-Luther-University Halle-Wittenberg and is in compliance with the Helsinki Declaration.

**Table 1:** Clinical and histomorphological data

Parameter	Total (n = 124)	OPN-a mRNA expression		OPN-b mRNA expression		OPN-c mRNA expression	
		
		low (n = 62)	high (n = 62)	low (n = 62)	high (n = 62)	low (n = 62)	high (n = 62)
*Sex*		**P = 0.02**		P = 0.10		P = 0.37	
Male	56	35	21	33	23	31	25
Female	68	27	41	29	39	31	37

*Histological subtype*		P = 0.96		P = 0.17		P = 0.17	
Liposarcoma	27	14	13	14	13	13	14
MFH/Fibrosarcoma	38	17	21	14	24	14	24
NS	15	8	7	9	6	9	6
RMS + LMS	29	15	14	14	15	15	14
other STS	15	8	7	11	4	11	4

*Tumor grade*		**P = 0.03**		**P = 0.05**		**P = 0.03**	
I	16	6	10	6	10	4	12
II	70	42	28	42	28	42	28
III	37	13	24	14	23	16	21

*Tumor stage*		P = 0.37		P = 0.43		P = 0.33	
I	15	7	8	7	8	5	10
II	57	33	24	33	24	33	24
III	40	18	22	17	23	19	21
IV	12	4	8	5	7	5	7

*Tumor localization*		P = 0.29		P = 0.26		P = 0.30	
Extremities	79	37	42	37	42	36	43
Thorax	12	5	7	5	7	6	6
Head and neck	4	1	3	1	3	1	3
Abdominal	27	18	9	17	10	18	9
Other	2	1	1	2	0	1	1

*Recurrence*		P = 1.00		P = 1.00		P = 1.00	
Yes	55	28	27	28	27	27	28
No	69	34	35	34	35	35	34

*Lymph node status*		P = 0.21		P = 0.21		**P = 0.03**	
N0	118	61	57	61	57	62	56
N1	6	1	5	1	5	0	6

*Distant metastases*		P = 0.28		P = 0.88		P = 0.88	
M0	45	25	20	23	22	23	2
M1	50	20	30	24	26	24	26
Evaluation not possible	3	2	1	2	1	2	1

*Radiation*		P = 1.00		P = 0.86		P = 0.86	
Yes	58	29	29	30	28	30	28
No	66	33	33	32	34	32	34

*Chemotherapy*		P = 0.74		P = 0.74		P = 0.74	
Yes	10	4	6	4	6	4	6
No	114	58	56	58	56	58	56

Abbreviations: MFH - malignant fibrous histiocytoma, NS - neurogenic sarcoma, RMS - rhabdomyosarcoma, LMS - leiomyosarcoma

### RNA preparation, cDNA synthesis and transcript analysis by quantitative real-time PCR (qRT-PCR)

The total RNA of the frozen tissue samples was isolated by the Trizol method (Invitrogen, Karlsruhe, Germany), and the cDNA was prepared using the RevertAid™ H Minus First Strand cDNA Synthesis Kit (Fermentas, St.Leon-Rot, Germany) according to the manufacturer's instructions. All qRT-PCR reactions were performed on a Rotorgene RG-6000 (LTF, Wasserburg, Germany) using the QuantiTect SYBRGreen PCR Kit (Fermentas). The primer sequences of the OPN splice variants and specific annealing temperatures are summarized in Table 2 (previously described in [14]). HPRT (hypoxanthine-guanine phosphoribosyltransferase) was used as a housekeeping gene (for standardization) and a marker for integrity of the cDNA. All methods were previously described in detail [8].

**Table 2:** Primers for quantitative real-time RT-PCR

Gene	Primer	Sequence 5' → 3'		Localization	Annealing temperature
HPRT	HPRT309	5'-TTGCTGACCTGCTGGATTAC-3'	sense	391-410	58°C
		
	HPRT507	5'-CTTGCGACCTTGACCATCTT-3'	antisense	633-652	

OPN-a	OPN-a fw 323	5'-ATCTCCTAGCCCCACAGAAT-3'	sense	323-342	58°C
		
	OPN-a rev 508	5'-CATCAGACTGGTGAGAATCATC-3'	antisense	529-508	

OPN-b	OPN-b fw 323	5'-ATCTCCTAGCCCCACAGAC-3'	sense	323-341	62°C
		
	OPN-b rev 509	5'-AAAATCAGTGACCAGTTCATCAG-3'	antisense	531-509	

OPN-c	OPN-c fw 246	5'-TGAGGAAAAGCAGAATGCTG-3'	sense	246-265	58°C
		
	OPN-c rev 377	5'-GTCAATGGAGTCCTGGCTGT-3'	antisense	396-377	

Sequences and localization of primers used in this study correspond to mRNA sequences of HPRT [Genbank: NM_000194.2], OPN-a [Genbank: NM_001040058.1], OPN-b [Genbank: NM_000582.2] and OPN-c [Genbank: NM_001040060.1]

### Statistical analysis

The statistical analysis was carried out using SPSS 17.0 (SPSS Inc., Chicago, IL, USA). Spearman's rho test was used for bivariate linear regression analyses. For survival analysis, the Kaplan-Meier method and the multivariate Cox's proportional hazard regression model were applied with appropriate adjustment to tumor type, tumor stage and tumor localization. We used a log-rank test to compare the survival curves of Kaplan-Meier analysis and to test for statistical differences. A two-tailed Fisher's exact-test or chi-square test was performed to determine the associations between mRNA expression level of OPN splice variants and different clinical parameters. A Wilcoxon signed-rank test was used to analyze the statistical differences of the 15 paired tumor and normal tissues. For survival analysis and comparison with clinical parameters, the cut-off values were set according to the median of mRNA expression levels of OPN splice variants (Table 3). A probability ≥ 95% (P ≤ 0.05) was considered an indicator of a significant difference between mean values.

**Table 3:** Kaplan-Meier analyses and multivariate Cox's regression analyses

STS patients	OPN splice variant	n	Median values [copies OPN splice variant mRNA/ copies HPRT mRNA]	Kaplan-Meier analysis		multivariate Cox's regression model		
				Survival [months]	P	RR	P	CI
All	OPN-a low	62	0.68	90				
	
	OPN-a high	62		71	1.7	1.7	0.06	1.0-3.0
	
	OPN-b low	62	0.49	117				
	
	OPN-b high	62		66	0.13	**2.1**	**0.02**	1.2-3.6
	
	OPN-c low	62	0.08	94				
	
	OPN-c high	62		81	0.38	**1.8**	**0.04**	1.0-3.1

Female	OPN-a low	34	0.83	116				
	
	OPN-a high	34		53	0.08	**3.0**	**0.01**	1.3-6.8
	
	OPN-b low	34	0.58	102				
	
	OPN-b high	34		42	0.13	**3.4**	**< 0.01**	1.4-8.2
	
	OPN-c low	34	0.09	92				
	
	OPN-c high	34		46	0.50	2.3	0.07	0.9-5.4

RT patients	OPN-a low	26	0.65	80				
	OPN-a high	26		76	**0.04**	3.5	0.07	0.9-13.0
	
	OPN-b low	26	0.42	147				
	OPN-b high	26		55	**0.05**	**10.3**	**< 0.01**	2.0-53.7
	
	OPN-c low	26	0.07	147				
	OPN-c high	26		55	**0.05**	**11.4**	**< 0.01**	2.2-59.3

Association of the mRNA expression of OPN splice variants in different groups of patients (all STS patients, females and RT patients) with overall survival. The median values of OPN-a, OPN-b and OPN-c were used as cut-off points to divide the STS patients into two groups: one with high and one with low (reference group) OPN mRNA levels in tumor tissues

## Results and discussion

### mRNA expression levels of OPN splice variants in STS tissues

The analysis of mRNA expression levels of OPN splice variants in the tumor tissues of 124 STS patients reveals a median transcript ratio of 0.68 copies OPN-a mRNA/copies HPRT mRNA (range: 9.30*10^-4^-85.29), 0.49 copies OPN-b mRNA/copies HPRT mRNA (range: 0.00-17.64) and 0.082 copies OPN-c mRNA/copies HPRT mRNA (range: 0.00-4.03). Comparison of the OPN splice variants mRNA expression levels clearly showed that OPN-a and OPN-b are expressed at a distinctly higher level than OPN-c (both with P < 0.01). The lower expression level of OPN-c is consistent with the findings in hepatocellular carcinoma, breast cancer and mesothelioma [12,15,16]. Furthermore, using bivariate linear Spearman-Rho correlation, we found a significant correlation of the mRNA expression levels between all OPN splice variants (r = 0.84-0.95, all with P < 0.01). Additionally, the mRNA expression level of total OPN and OPN splice variants show a weak correlation (n = 65, OPN-a: r = 0.32, P < 0.01; OPN-b: r = 0.30, P = 0.02; OPN-c: r = 0.26, P = 0.04).

**Figure 1:**
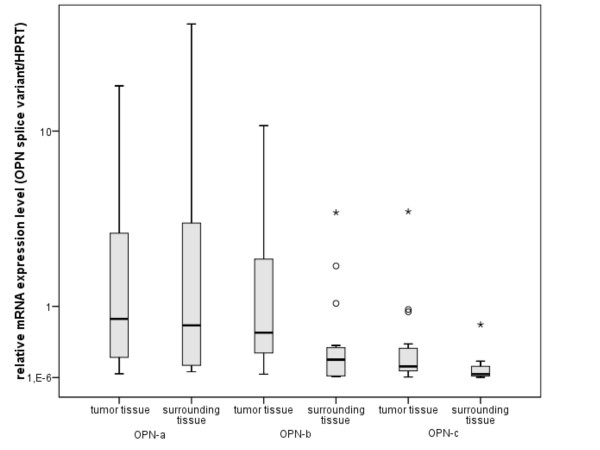
**Comparison of mRNA expression of OPN splice variants in surrounding and tumor tissue**. The boxplot shows the relative mRNA expression levels of OPN-a, OPN-b and OPN-c in tumor and paired surrounding tissues of 15 STS patients. The boxplot displays the median value of each data set, which is indicated by the centerline. The edges of the boxes represent the 25th percentile and 75th percentile. The 10th and 90th percentiles are marked through the horizontal lines outside the boxes. Circles and asterisks mark outliers (1.5 to 3 box lengths from the edge of the boxes) and far outliers (more than 3 lengths from the edge of the boxes).

In surrounding tissues of 15 STS patients we calculated a median transcript ratio of 0.66 copies OPN-a mRNA/copies HPRT mRNA (range: 5.80*10^-2^-30.19), 0.19 copies OPN-b mRNA/copies HPRT mRNA (range: 7.20*10^-3^-3.98) and 0.034 copies OPN-c mRNA/copies HPRT mRNA (range: 9.55*10^-4^-0.67). The median mRNA expression levels of OPN splice variants reveal that OPN-b and OPN-c in paired tumor tissues are expressed on a higher level compared to surrounding tissues (n = 15, p = 0.07 and p = 0.06). Figure 1 demonstrates a 2.9 and 3.4-fold increased mRNA expression level of OPN-b and OPN-c in tumor tissues. However, OPN-a mRNA expression level was not increased in tumor tissues (1.2-fold, p = 0.80). Several other studies confirm in tumor tissues a higher mRNA expression level of OPN splice variants than in surrounding or normal tissues [19,20]. In agreement with our analysis, in breast cancer and ovarian cancer, especially OPN splice variants OPN-b and OPN-c are expressed on elevated mRNA levels compared to surrounding or normal tissues [10,14,15,18].

### Bivariate linear analyses of mRNA expression levels of OPN splice variants with clinical and histomorphological parameters

Examinations of the relation of mRNA expression levels of OPN splice variants with clinical and histomorphological parameters revealed a significant correlation of the mRNA expression level of OPN-a with sex and tumor grade (Table 1). More male patients have a low OPN-a mRNA expression level compared to female patients (P = 0.02, Table 1). Grade 3 tumors are strongly associated with high mRNA expression levels of OPN-a, OPN-b and OPN-c (P = 0.03, P = 0.05 and P = 0.03, respectively). The mRNA expression levels of the OPN splice variants were also significantly higher in high-grade gliomas than in low-grade gliomas [9,17]. Recently, Patani *et al. *(2008) verified that the mRNA expression levels of OPN-b and OPN-c were also increased with higher tumor grade in breast cancer. Furthermore, we found that all patients with lymph node metastases had a significantly higher OPN-c mRNA expression level (P = 0.03). It is well known that OPN-c mediates anchorage independence and tumor invasion [10,21], which are crucial processes for the development of metastases. Several studies showed that OPN-c is expressed at higher levels in invasive tumor cells compared to noninvasive tumor cells [13,14,17,21]. Furthermore, it has been demonstrated in these studies that OPN-c influences the expression of several migration/invasion markers, such as MMP-2, MMP-9 and uPA, which promote tumor cell invasion. This is in accordance with the correlation we found between the OPN splice variants, the uPA, uPAR and PAI mRNA and the protein expression levels in STS (data not shown). Previously, we could show that the protein levels of uPA, uPAR and PAI in tumor tissue and serum are also associated with poor prognosis of STS patients [22]. However, we found no correlation between the mRNA expression level of OPN splice variants and the tumor stage, histological tumor subtype (Additional file 1), tumor localization or recurrence.

### mRNA expression levels of OPN splice variants and disease-associated survival

**Figure 2:**
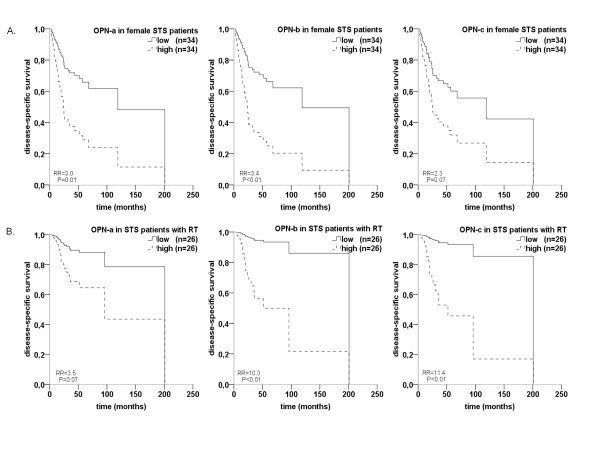
**Multivariate Cox's regression hazard analysis: Association of OPN splice variant mRNA expression levels with disease-specific survival of STS patients**. Cox's proportional hazard regression models were adjusted to tumor stage, tumor entity, tumor localization and the expression of OPN-a, OPN-b or OPN-c for 68 female STS patients (A.) and 52 STS patients that received radiotherapy (B.). The median values were used as cut-off points to divide the STS patients into two groups: one with high and one with low (reference group) OPN mRNA levels in tumor tissues. **A**. Increased expressions of OPN-a and OPN-b are strongly associated with a 3.0-fold and a 3.4-fold increased risk of tumor-related death in female STS patients, respectively (P = 0.01, CI = 1.3-6.8; P < 0.01, CI = 1.4-8.2). The STS patients with an increased OPN-c expression level have a 2.3-fold (P = 0.07, CI = 0.9-5.4) increased risk of tumor-related death. **B**. In STS patients that received radiotherapy, elevated mRNA expression level of OPN-b and OPN-c are significantly correlated with an increased risk of tumor-related death (RR = 10.3, P < 0.01, CI = 2.0-53.7; RR = 11.4, P < 0.01, CI = 2.2-59.3). RT patients with an increased OPN-a mRNA expression level have a 3.5-fold increased risk of tumor-related death, but this result was not significant (P = 0.07, CI = 0.9-13.0).

We performed multivariate Cox's proportional hazard regression analysis to study the correlation between OPN splice variant levels and tumor-specific survival. The STS patients with high mRNA expression levels of the three OPN splice variants have a worse prognosis than those with low mRNA expression levels in their tumors. An elevated mRNA expression level of OPN-b or OPN-c is significantly correlated with a 2.1-fold or 1.8-fold increased risk of tumor-related death for STS patients, respectively (P = 0.02, CI = 1.2-3.6; P = 0.04, CI = 1.0-3.1) (Table 3). The mRNA expression level of OPN-a shows an association trend with the prognosis of STS patients (RR = 1.7, P = 0.06) (Table 3). Additionally, STS patients had worst prognosis when all OPN splice variants are expressed on high mRNA levels (data not shown). A study on 15 adult STS patients found a significant increase of total OPN mRNA levels in tumor tissues compared to normal tissues [5]. Another study on 41 osteosarcoma patients reported that higher OPN mRNA levels were associated with a worse prognosis [23]. However, our preceding data of 68 STS patients revealed that mRNA expression level of whole OPN did not significantly correlate with prognosis [8]. Up to now, the prognostic relevance of the OPN splice variants was only determined for breast cancer patients. The breast cancer patients with high mRNA expression levels of OPN-b or OPN-c had significantly shorter survival times than those with low mRNA expression levels [15]. Because we found much lower mRNA expression levels of OPN-a in the tumors of male patients (n = 56) than in those of female patients (n = 68), we performed sex-specific survival analyses (Table 3 and Figure 2A.). A multivariate Cox's regression model also revealed that female STS patients have a significantly worse prognosis than male STS patients. The women with high OPN-a or OPN-b mRNA expression levels have a 3.0-fold (P = 0.01, CI = 1.3-6.8) or 3.4-fold (P < 0.01, CI = 1.4-8.2) increased risk of tumor-related death, respectively. The different prognostic impact of the mRNA expression level in men and women is possibly caused by hormonal regulation of mRNA expression by female sex hormones or hormone receptors [24].

Therapy of STS patients comprises surgical removal of the tumor and treatment with radio- and/or chemotherapy. To investigate the prognostic impact of the mRNA expression level of OPN splice variants prior to radiotherapy, we analyzed STS patients who received a curative radiotherapy (n = 52) (RT patients). In the Kaplan-Meier analysis, we found that the RT patients with low mRNA expression levels of all OPN splice variants have a significant survival benefit compared to those with high mRNA expression levels in their tumors (P < 0.05, Table 3). In addition multivariate Cox's regression models revealed that RT patients with high mRNA expression levels of OPN-b and OPN-c have a 10.3-fold and 11.4-fold increased risk of tumor-related death, respectively (P < 0.01, CI = 2.0-53.7; P < 0.01, CI = 2.2-59.3) (Figure 2B., Table 3). In the STS patients who did not receive radiotherapy (n = 62), the mRNA expression level of the OPN splice variants had no prognostic importance (data not shown). Consequently, the inhibiting of OPN could provide an additional survival benefit for STS patients who are treated with radiotherapy. Similarly, Overgaard *et al. *(2005) found that high OPN plasma concentrations are associated with a poor prognosis in patients with head and neck cancer after radiotherapy [25]. Our in vitro study verified the association of OPN and radiotherapy and proved that the inhibition of OPN mRNA expression increases the radiosensitivity of the mamma carcinoma cells [26].

## Conclusions

In the present study, we found for the first time that the mRNA expression levels of OPN-b and OPN-c were significantly correlated with the clinical outcome of STS patients. Our data demonstrate that female STS patients and RT patients with low mRNA expression levels of OPN splice variants have a distinct survival benefit. In fact, the different roles of the OPN splice variants in angiogenesis, cellular invasion, cancer progression, and metastasis are widely discussed in the literature [14,17,18,21,27]. Further studies are needed to clarify why OPN splice variants have different effects on the prognosis of cancer patients.

In summary, our data suggest that high expression levels of OPN splice variants are negative prognostic and predictive markers, particularly for female STS patients and those who receive curative radiotherapy. However, more data are necessary to evaluate the OPN splice variants in the clinical management of STS patients.

## Abbreviations

RR: Relative risk of tumor-related death; P: Probability; CI: 95% confidence interval; r: Correlation coefficient

## Competing interests

The authors declare that they have no competing interests.

## Authors' contributions

AH and HW designed the study, performed experimental procedures, analyzed the data and drafted the manuscript. TG, MKa, HT and DV aided in study design, analyzed the data and reviewed the manuscript. PW treated the patients, collected material and data and reviewed the manuscript. MKo performed experimental procedures, analyzed the data and reviewed the manuscript. MB designed the study, analyzed the data and drafted the manuscript. All authors read and approved the final manuscript.

## Pre-publication history

The pre-publication history for this paper can be accessed here:

http://www.biomedcentral.com/1471-2407/12/131/prepub

## Supplementary Material

Additional file 1 **Median mRNA expression level of the OPN splice variants in the different histotypes of soft tissue sarcoma**. Click here for file
