# Supplementary figures and images for: Prognostic impact of mRNA levels of osteopontin splice variants in soft tissue sarcoma patients
Source: BMC Cancer. 2012 Apr 2;12:131. doi: 10.1186/1471-2407-12-131 (PMC3364873; doi:10.1186/1471-2407-12-131)

## Slide 1
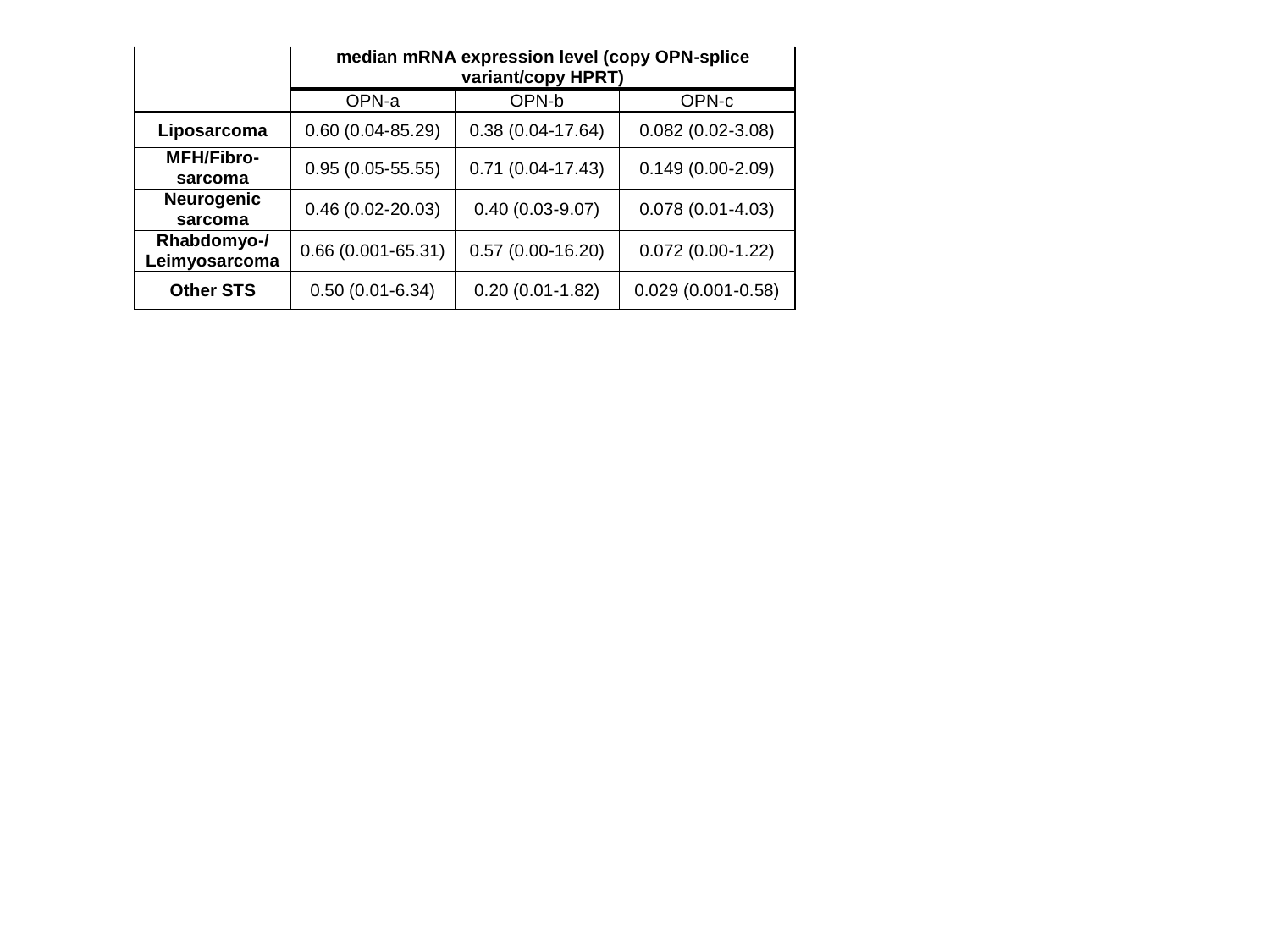

Supplement: Additional file 1 — Median mRNA expression level of the OPN splice variants in the different histotypes of soft tissue sarcoma. [file 1471-2407-12-131-S1.PPT]
